# Effects of Roughage Physically Effective Neutral Detergent Fiber Levels on Rumen Fermentation, Microbiota, and Metabolomic Profiles in Yaks

**DOI:** 10.3390/microorganisms14102298

**Published:** 2026-10-09

**Authors:** Long Ye, Dongwen Dai, Xun Wang, Zhilong Wang, Jiaying Lv, Cheng Tie, Jiyuan Zhang, Yijuan Ma, Shatuo Chai, Shuxiang Wang

**Affiliations:** 1 College of Agriculture and Animal Husbandry, Qinghai University, Xining 810016, China; 18427220433@163.com (L.Y.); 2025990090@qhu.edu.cn (D.D.); wangxun513@163.com (X.W.); 18225922403@139.com (Z.W.); lvjiaying1123@163.com (J.L.); mayijuan@qhu.edu.cn (Y.M.); 2 Qinghai Provincial Yak Engineering and Technology Research Center, Xining 810016, China; 3 Key Laboratory of Plateau Grazing Animal Nutrition and Feed Science of Qinghai Province, Qinghai University, Xining 810016, China; 4 Yak Engineering Technology Research Centre of Qinghai Province, Qinghai University, Xining 810016, China; 5 Xinghai County Animal Husbandry and Veterinary Station, Xinghai 813300, China; 19109713151@163.com

**Keywords:** peNDF, yak, growth performance, rumen fermentation, rumen microbiota, metabolomics

## Abstract

This study investigated the effects of three roughage physically effective neutral detergent fiber (peNDF_8.00_) levels (11.52%, 13.16%, and 15.43% on an as-fed roughage basis) on growth performance, rumen fermentation, serum biochemical parameters, rumen microbiota, and rumen metabolomic profiles in yaks. Thirty healthy yaks were allocated to three treatment groups (A, B, and C). The roughage peNDF_8.00_ treatments did not significantly affect short-term growth performance. Ruminal valerate concentration was significantly higher in group C than in group A (*p* < 0.05), while the molar proportions of isobutyrate and isovalerate differed significantly among treatments (*p* < 0.001). Serum aspartate aminotransferase (AST) activity and blood urea nitrogen (BUN) concentration also differed significantly among treatments (*p* < 0.05). Rumen microbiota analysis revealed treatment-associated differences in Chao1 richness and variation in microbial community structure, while LEfSe identified Bacteroidota and Xylanibacter as exploratory candidate biomarkers of group C. PICRUSt2 analysis suggested treatment-associated variation in predicted microbial functional potential. Untargeted metabolomics identified multiple candidate treatment-associated metabolic features; however, no KEGG pathway remained significant after Benjamini–Hochberg false discovery rate (FDR) correction. Overall, higher roughage peNDF_8.00_ was associated with variation in rumen fermentation, microbial community structure, and metabolomic profiles, without a detectable effect on short-term growth performance.

## 1. Introduction

Yaks (Bos grunniens) are indigenous ruminants of the Qinghai–Tibetan Plateau and support local livelihoods by providing meat, milk, fiber, and draft power [1,2]. Long-term adaptation to alpine environments has been accompanied by physiological and rumen microbial characteristics that facilitate survival under low temperatures, hypoxia, and seasonal forage limitation [3]. Comparative studies indicate that the rumen microbiomes of yaks and cattle differ in their responses and stability under plateau conditions [4]. Traditional yak production relies heavily on natural grazing, but grassland degradation and seasonal forage shortages can constrain nutrient supply [1,5]. Supplementary feeding and intensive feeding systems have therefore been investigated as strategies to improve cold-season nutrition and production [6,7].

Physically effective neutral detergent fiber (peNDF) integrates dietary fiber concentration with feed physical structure and is widely used to characterize the physical effectiveness of ruminant diets [8,9]. The physical effectiveness of dietary fiber can influence chewing and rumination, salivary buffering, ruminal pH, rumen mat formation, and digesta passage [8,9,10,11]. In dairy cows fed high-concentrate diets, increasing peNDF has been associated with greater chewing activity, changes in rumen fermentation, and alterations in plasma metabolites and milk production [12]. Studies using corn silage fractions separated with the Penn State Particle Separator (PSPS) further demonstrate that particle-size fractions differ in physical effectiveness [13]. The PSPS was originally described by Lammers et al. [14]. Most mechanistic evidence regarding peNDF has been generated in cattle, particularly dairy cattle [13].

Information on peNDF in yaks remains limited. Given the yak-specific rumen ecology associated with adaptation to high-fiber plateau diets [3,4], responses to feed physical structure may not fully parallel those reported in cattle under conventional production conditions. Few studies have jointly evaluated growth performance, rumen fermentation, microbial community structure, and rumen metabolomics in yaks subjected to a peNDF gradient. In the present experiment, roughage physical effectiveness was altered by changing oat-hay particle size while maintaining the same ingredient formulation and concentrate allowance. The three roughage peNDF_8.00_ levels were generated by applying three degrees of oat-hay particle-size processing and were selected to represent low, intermediate, and high roughage physical-effectiveness treatments within the same feeding regimen. This design has practical relevance to housed yak feeding because roughage physical structure can be modified through forage processing without changing ingredient inclusion rates. The reported roughage peNDF_8.00_ values are as-fed treatment descriptors derived from the NDF concentration and particle-size distribution of the roughage component; they should not be interpreted as peNDF_8.00_ concentrations of the complete consumed diet, as established requirement values, or as defining an optimal range for yaks. Therefore, this study evaluated the associations of three roughage peNDF_8.00_ treatments with growth performance, serum biochemical indices, rumen fermentation, rumen microbiota, and rumen metabolomic profiles in yaks.

## 2. Materials and Methods

### 2.1. Experimental Animals and Experimental Design

The experiment was conducted from mid-January to March 2025 at Hengyuan Animal Husbandry Breeding Professional Cooperative in Xinghai County, Qinghai Province, China. The mean outdoor air temperature in Xinghai County from January to March 2025 was −7.2 °C. Thirty healthy, 36-month-old, castrated male yaks with similar initial body weights and body condition were selected and allocated to three dietary treatment groups (A, B, and C) based on comparable mean body weight, with 10 yaks per group. All animals were housed and fed in group pens; they were not individually fed. The experiment lasted for 70 days, including a 10-day adaptation period followed by a 60-day experimental period.

The dietary treatments were designed to establish different feed physical structures by modifying oat-hay particle size while keeping ingredient composition and inclusion rates unchanged. Oat-hay particle length was altered by varying the number of grinding passes, after which the processed hay was mixed with corn silage. Particle-size distribution was determined with the Penn State Particle Separator according to Lammers et al. [14]. The resulting roughage peNDF_8.00_ treatment labels were 11.52%, 13.16%, and 15.43% on an as-fed roughage basis for groups A, B, and C, respectively; the reporting basis is detailed in Section 2.2.

During the experimental period, the roughage diet was offered ad libitum at 08:00 h each day. At 16:00 h, concentrate was provided to all groups at a planned allowance of 2.5 kg/yak/day, and clean drinking water was available ad libitum throughout the experiment. Because the concentrate was supplied separately from the roughage, it was not included in the PSPS particle-size measurement or in the calculation of roughage peNDF_8.00_. The ingredient and chemical composition of the experimental diet are presented in Table 1. Diet formulation and nutrient evaluation were conducted with reference to NASEM (2016) [15]. Dry matter, crude protein, ether extract, and ash were determined according to AOAC International (2019) [16], whereas neutral detergent fiber and acid detergent fiber were analyzed following Van Soest et al. (1991) [17].

### 2.2. Determination of Roughage peNDF

The roughage peNDF_8.00_ treatment gradient was characterized using the PSPS method of Lammers et al. [14] within the physically effective fiber framework proposed by Mertens [8]. Roughage samples were separated by particle size, and the physical effectiveness factor (pef_8.00_) was calculated as the proportion of roughage dry matter retained on the >19.0 and 8.0–19.0 mm sieves. The NDF concentration used for the peNDF_8.00_ calculation was measured in the same roughage mixture that was subjected to particle-size separation, rather than in the complete diet including the separately supplied concentrate. The roughage mixture contained 42.31% NDF on a DM basis (Table 1), equivalent to 17.43% NDF on an as-fed roughage basis. Roughage peNDF_8.00_ was calculated as roughage NDF on an as-fed basis × roughage pef_8.00_, yielding 11.52%, 13.16%, and 15.43% for groups A, B, and C, respectively(Table 2). Because the concentrate allowance was provided separately and was not included in the particle-size analysis, these values represent the peNDF_8.00_ of the roughage component and should not be interpreted as the peNDF_8.00_ concentration of the complete consumed diet. Unless otherwise specified, peNDF_8.00_ values reported throughout the manuscript refer to the roughage component.

### 2.3. Sample Collection

The formal experimental period lasted 60 d. All yaks were weighed under fasting conditions before morning feeding at the beginning and end of the trial to obtain the initial body weight (IBW) and final body weight (FBW), respectively. Total weight gain (TWG) was calculated as the difference between FBW and IBW, and average daily gain (ADG) was determined by dividing TWG by the duration of the experimental period.

On the final day of the trial, blood samples were collected from all yaks via jugular venipuncture before morning feeding. The samples were kept at room temperature for 2 h and then centrifuged at 1095× *g* for 10 min at 4 °C. The separated serum was transferred into 2 mL cryovials and stored at −80 °C until biochemical analyses were performed.

On the same day, five yaks were randomly selected from each treatment group for rumen fluid sampling, providing five biological replicates per group and a total of 15 samples for volatile fatty acid (VFA), microbiome, and metabolomic analyses. Approximately 50 mL of rumen fluid was obtained from each animal using an oral rumen-fluid sampling tube coupled with a sterile syringe. To minimize potential salivary contamination during oral sampling, the first 20 mL of fluid collected was discarded. Previous studies have shown that rumen fluid obtained using an oral stomach tube may be affected by salivary contamination and the depth of tube insertion [18]. The remaining rumen fluid was immediately filtered through four layers of sterile gauze, aliquoted into 2 mL cryovials, and rapidly stored in liquid nitrogen. All rumen fluid samples were collected once before morning feeding on the final day of the experiment.

### 2.4. Determination of Volatile Fatty Acids

Rumen volatile fatty acids (VFAs) were quantified by gas chromatography with flame ionization detection (GC-FID). Sample processing was adapted, with minor modifications, from a previously reported procedure for VFA determination in yak rumen fluid [19]. After thawing at 4 °C, the rumen fluid was centrifuged at 12,000× *g* for 10 min at 4 °C. One milliliter of supernatant was collected and acidified with 0.2 mL of 25% metaphosphoric acid containing 2-ethylbutyric acid as the internal standard. The preparation was mixed thoroughly by vortexing and held at 4 °C for 30 min to allow protein precipitation. A second centrifugation was then performed under the same conditions (12,000× *g*, 10 min, 4 °C). Before injection into the gas chromatograph, the resulting supernatant was passed through a 0.22 μm membrane filter.

Chromatographic measurements were performed on an Agilent 7890B system (Agilent Technologies, Santa Clara, CA, USA) equipped with a flame ionization detector and a DB-FFAP capillary column (30 m × 0.25 mm × 0.25 μm). Nitrogen was used as the carrier gas. The injector temperature was maintained at 250 °C and the detector at 280 °C. For chromatographic separation, the oven temperature was initially set at 100 °C and maintained for 1 min. It was then raised to 150 °C at 10 °C/min, followed by a further increase to 220 °C at 20 °C/min. The final temperature was held for 5 min. A 1 μL aliquot of each prepared sample was introduced into the system using split injection.

The six VFAs evaluated were acetic acid, propionic acid, isobutyric acid, butyric acid, isovaleric acid, and valeric acid. Compound identity was confirmed by matching chromatographic retention times with those obtained from the corresponding external standards. Concentrations were calculated from calibration curves using 2-ethylbutyric acid for internal-standard correction. The total VFA concentration was calculated as the sum of the concentrations of the six quantified VFAs. For each biological sample, the molar proportion of each individual VFA was calculated as its concentration divided by the summed concentration of the six VFAs × 100. The acetate-to-propionate ratio was calculated from the corresponding sample-level acetate and propionate concentrations. Both absolute VFA concentrations and derived molar proportions, together with the acetate-to-propionate ratio, were included in the subsequent statistical analyses.

### 2.5. Serum Biochemical Indices

Serum concentrations of calcium (Ca), phosphorus (P), albumin (ALB), glucose (GLU), total protein (TP), cholesterol (CHOL), blood urea nitrogen (BUN), and triglycerides (TG), as well as the activities of lactate dehydrogenase (LDH), alkaline phosphatase (ALP), alanine aminotransferase (ALT), and aspartate aminotransferase (AST), were determined using an automatic biochemical analyzer with commercial diagnostic kits (Shenzhen Mindray Bio-Medical Electronics Co., Ltd., Shenzhen, China) according to the manufacturer’s instructions and standard veterinary clinical biochemistry procedures [20,21].

Serum Ca and P concentrations were determined using colorimetric assays, TP was measured by the biuret method, and ALB was quantified using the bromocresol green method. Serum GLU, CHOL, TG, and BUN concentrations were analyzed using enzymatic colorimetric assays, whereas the activities of LDH, ALP, ALT, and AST were determined by kinetic spectrophotometric methods [22]. Calibration standards and quality-control sera were included during the analytical procedures to ensure analytical accuracy and reproducibility. All samples were analyzed in duplicate, and the mean values were used for statistical analysis. All assays were performed under the same analytical conditions to minimize inter-assay variation.

### 2.6. Rumen Microbiota Analysis

Total microbial DNA was isolated from rumen fluid using a cetyltrimethylammonium bromide (CTAB)-based procedure adapted from previously published methods [23,24]. The integrity and quality of the extracted DNA were assessed by 1.0% agarose gel electrophoresis together with spectrophotometric measurements of DNA concentration and purity. Samples meeting the quality requirements were normalized to 1 ng/μL with sterile water and subsequently used for amplification.

The bacterial V3–V4 region of the 16S rRNA gene was targeted using the barcoded primer pair 343F (TACGGRAGGCAGCAG) and 798R (AGGGTATCTAATCCT). Amplification reactions were prepared in a final volume of 25 μL containing DNA template, forward and reverse primers, PCR master mix, and nuclease-free water. Thermal cycling began with denaturation at 94 °C for 5 min, followed by 30 cycles consisting of 30 s at 94 °C, 30 s at 50 °C, and 60 s at 72 °C. A final extension was carried out at 72 °C for 7 min.

The resulting amplicons were examined on a 1.0% agarose gel and purified using a MinElute Gel Extraction Kit (Qiagen, Hilden, Germany). Purified PCR products were then combined in equal quantities for preparation of paired-end sequencing libraries. Library construction and subsequent sequencing were conducted by OE Biotech Co., Ltd. (Shanghai, China).

Raw paired-end reads were processed with Cutadapt [25] to remove primer sequences and then analyzed using QIIME 2 (version 2020.11) [26]. Quality filtering, denoising, paired-end merging, chimera removal, and amplicon sequence variant (ASV) inference were performed using DADA2 version 1.18.0 through the q2-dada2 plugin [27]. According to the archived sequencing-service report, the QIIME 2/DADA2 workflow used default parameters; the parameters that could be confirmed were trim-left-f/r = 0, max-ee-f/r = 2.0, trunc-q = 2, minimum overlap = 12 nt, independent pooling, and consensus chimera removal. Read-specific truncation lengths were not retained in the archived report. Taxonomic annotation of representative ASV sequences was performed against the SILVA ribosomal RNA gene database (release 138.2) [28]. Before diversity analyses, the ASV table was rarefied to an even sequencing depth of 24,808 reads per sample. Alpha-diversity indices, including Chao1 and Shannon, were calculated from the ASV table, and beta diversity was assessed using Bray–Curtis distances and visualized by principal coordinate analysis (PCoA). Microbial functional profiles were predicted using PICRUSt2 (version 2.3.0b0) [29]. Across the 15 samples, 78,006–81,613 raw reads were generated per sample; 32,476–55,810 merged clean tags were retained after filtering, denoising, and paired-end merging; and 32,265–50,648 valid tags remained after chimera removal. Rarefaction curves generally approached plateaus, and Good’s coverage values were close to 1.0, indicating that sequencing depth was broadly adequate for diversity analyses. The archived sequencing dataset contained the 15 biological rumen samples but did not include a separately sequenced extraction/PCR negative-control library; therefore, low-abundance taxa should be interpreted cautiously.

### 2.7. Rumen-Fluid Metabolomic Analysis

Rumen-fluid metabolite profiles were characterized using an untargeted ultra-high-performance liquid chromatography–high-resolution mass spectrometry (UHPLC-HRMS) platform. Sample preparation was adapted from a previously reported rumen-fluid metabolomics protocol [30]. Frozen samples were thawed on ice, and an aliquot of each sample was mixed with a pre-cooled methanol/acetonitrile/water solution to extract metabolites and precipitate proteins. After vortexing and sonication in an ice-water bath, the mixtures were subjected to high-speed centrifugation at 4 °C. The resulting supernatants were collected and filtered through 0.22 μm membranes before instrumental analysis. A pooled quality-control (QC) sample was prepared by combining equal aliquots of all sample extracts and was injected throughout the analytical sequence to monitor signal stability and analytical reproducibility [31,32]. QC reproducibility was evaluated separately in positive- and negative-ion modes. Pairwise Pearson correlation coefficients were calculated among three replicate injections of the pooled QC sample (QC1–QC3), and QC clustering was further evaluated by unsupervised principal component analysis (PCA). Metabolites with a coefficient of variation (CV) < 30% across the pooled QC samples were retained for downstream analysis.

Chromatographic separation was carried out on a Vanquish UHPLC system (Thermo Fisher Scientific, Waltham, MA, USA) equipped with an ACQUITY UPLC HSS T3 column (2.1 mm × 100 mm, 1.8 μm; Waters, Milford, MA, USA). The column temperature and flow rate were maintained at 40 °C and 0.30 mL/min, respectively. Water containing 0.1% formic acid was used as mobile phase A, whereas acetonitrile containing 0.1% formic acid served as mobile phase B. The gradient was programmed as follows: 5% B from 0 to 1 min, 5–95% B from 1 to 9 min, 95% B from 9 to 12 min, 95–5% B from 12 to 12.1 min, and 5% B from 12.1 to 15 min for column re-equilibration. The injection volume was 1.00 μL. Mass-spectrometric detection was performed using a Q Exactive Orbitrap mass spectrometer (Thermo Fisher Scientific, Waltham, MA, USA) coupled to an electrospray ionization source operating in both positive- and negative-ion modes. Spray voltages were set at 3.5 kV and 2.8 kV for positive and negative ionization, respectively, with the capillary temperature maintained at 320 °C. Sheath and auxiliary gas flow rates were 35 and 10 arb, respectively. Full-scan spectra were acquired over an m/z range of 70–1050 at a resolution of 70,000, while the MS/MS resolution was set to 17,500.

Raw UHPLC-MS data were processed using XCMS for peak detection, peak integration, alignment, and retention-time correction. Peak areas were normalized to the total peak area. Metabolite annotation was based on accurate mass, retention behavior, and MS/MS fragmentation patterns by comparison with public and in-house databases, including HMDB [33], METLIN [34], and KEGG [35]. For multivariate analysis, the metabolomic data were log-transformed and standardized prior to PCA. Unsupervised PCA was performed separately for positive- and negative-ion modes using both biological and pooled QC samples to evaluate overall metabolic variation, sample distribution, and QC clustering without using treatment-group information. Partial least squares discriminant analysis (PLS-DA) was subsequently applied as a supervised approach to assess treatment-associated metabolic differences. PLS-DA model performance was evaluated using R^2^Y and Q^2^Y values, and potential overfitting was further examined by permutation testing, with the corresponding results provided in the Supplementary Materials. Candidate differential metabolic features were initially screened using the predefined criteria of variable importance in projection (VIP) > 1.0, FC > 1.5 or FC < 0.667, and nominal *p* < 0.05. To account for multiple testing at the metabolite level, raw *p* values for all 3026 tested metabolic features (1842 positive-ion and 1184 negative-ion features) were adjusted using the Benjamini–Hochberg false discovery rate (FDR) procedure within each pairwise comparison. Features with an FDR-adjusted *p* value (q value) < 0.05 were considered statistically significant after multiple-testing correction. The complete metabolite-level results, including raw and FDR-adjusted *p* values, are provided in the Supplementary Materials. KEGG pathway enrichment analysis was subsequently conducted to identify pathway-level signals associated with the observed metabolic differences [35].

### 2.8. Statistical Analysis

Statistical analyses of growth performance, ruminal VFA concentrations and molar proportions, the acetate-to-propionate ratio, and serum biochemical parameters were conducted using SPSS version 27.0 (IBM Corp., Armonk, NY, USA). Results are expressed as mean ± standard error of the mean (SEM). The distributional normality of the data and equality of variances were examined using the Shapiro–Wilk and Levene tests, respectively. When both assumptions were satisfied, differences among the three treatment groups were evaluated using one-way analysis of variance (ANOVA). When the overall ANOVA was significant, pairwise comparisons among treatment means were performed using Tukey’s honestly significant difference (HSD) test. For variables that did not satisfy the assumptions for parametric analysis, differences among the three treatment groups were evaluated using the Kruskal–Wallis test; when appropriate, pairwise comparisons were subsequently performed using Dunn’s test with Holm adjustment for multiple comparisons. Although the three roughage peNDF_8.00_ treatments represented an ordered quantitative gradient (11.52%, 13.16%, and 15.43%), the post-hoc analyses were used only for pairwise mean separation among the predefined treatment groups and were not interpreted as evidence of a linear or monotonic dose–response relationship. Statistical significance was defined as *p* < 0.05.

**Figure 1:**
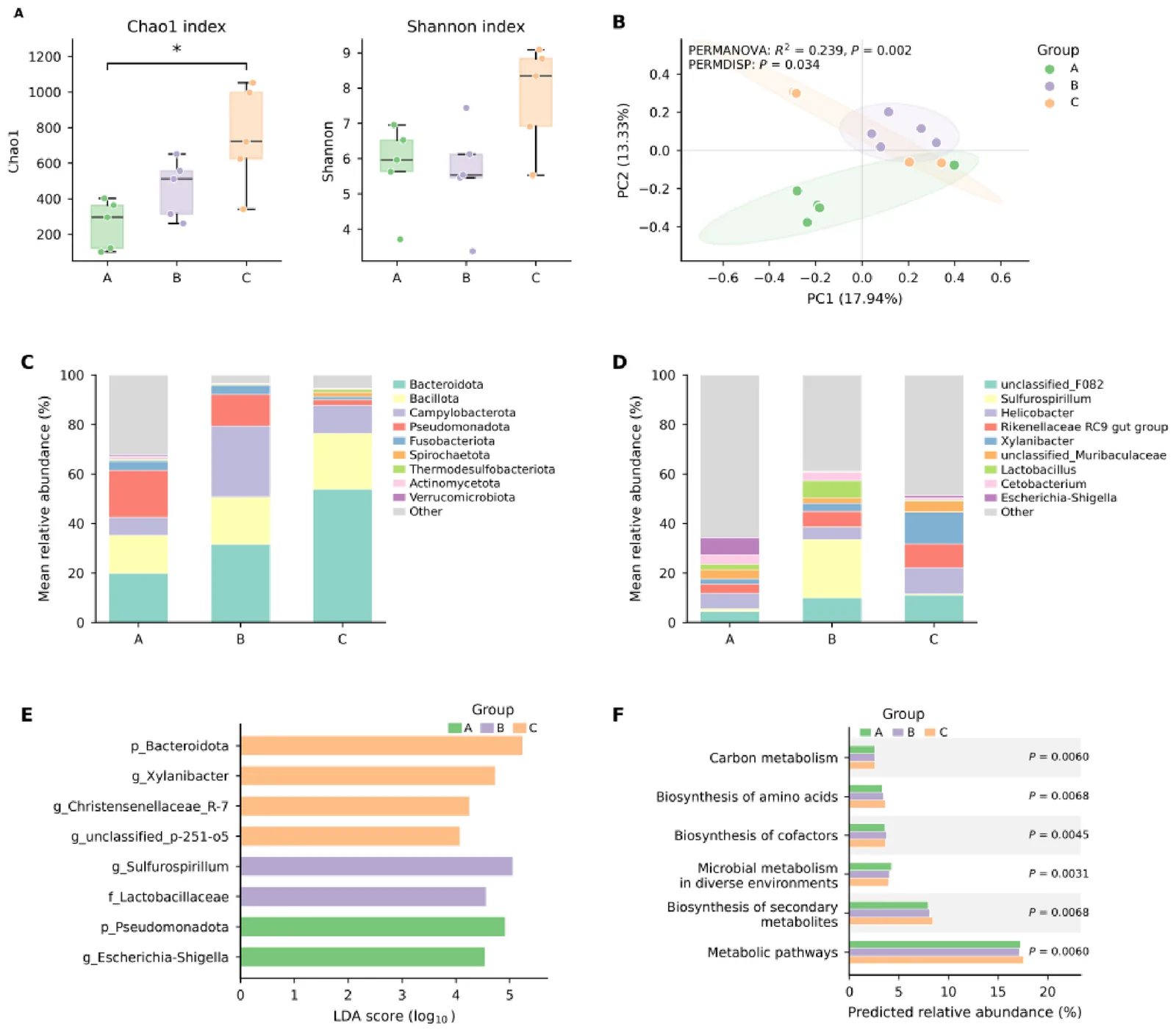
Effects of different roughage peNDF levels on the rumen microbial community of yaks (*n* = 5 biological replicates per treatment): (**A**) α-diversity analysis of Chao1 and Shannon indices; Chao1 was higher in group C than in group A after Dunn’s test with Holm adjustment (adjusted *p* = 0.0267), whereas Shannon did not differ significantly among groups (Kruskal–Wallis *p* = 0.134). The asterisk (*) indicates a significant pairwise difference based on Dunn’s test with Holm adjustment (adjusted *p* < 0.05). (**B**) β-diversity analysis based on PCoA using Bray–Curtis distances; PERMANOVA was used to evaluate overall treatment-associated differences, and PERMDISP was used to assess homogeneity of within-group multivariate dispersion; (**C**) top 10 phyla by relative abundance; (**D**) top 10 genera by relative abundance; (**E**) exploratory LEfSe analysis using LDA score > 4 and nominal *p* < 0.05 without FDR correction; (**F**) exploratory PICRUSt2-based prediction of microbial functional potential for selected KEGG Level 3 pathways, with nominal Kruskal–Wallis *p* values shown.

**Figure 2:**
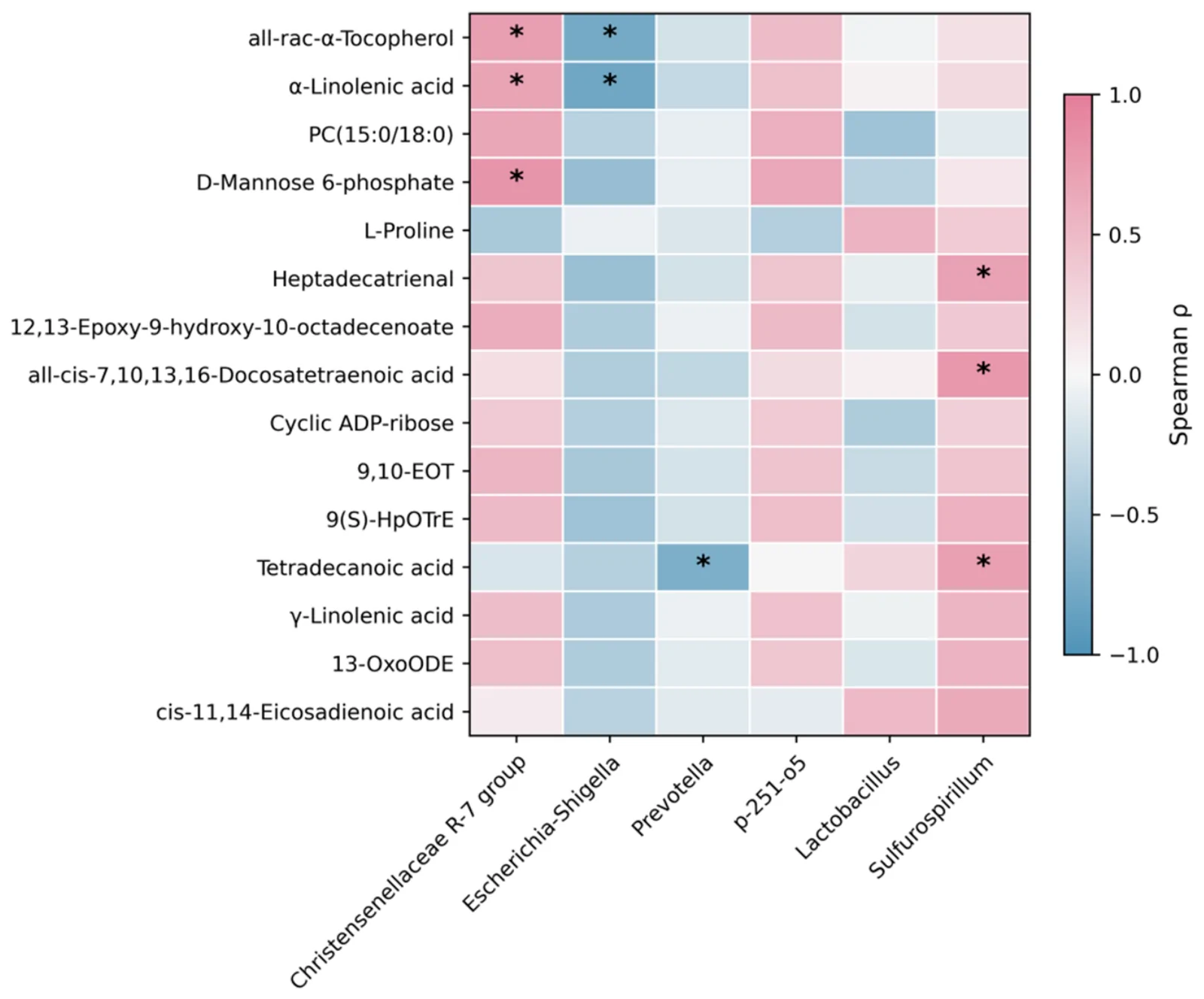
Spearman rank correlations between selected rumen microbial taxa and candidate metabolites across 15 paired biological samples (*n* = 5 per treatment). Colors represent Spearman’s ρ. The p values from all 90 microbe–metabolite comparisons were adjusted using the Benjamini–Hochberg false discovery rate (FDR) procedure. Asterisks (*) indicate correlations that remained significant after FDR correction (q < 0.05).

For rumen microbiota data, alpha-diversity indices were first evaluated among groups using the Kruskal–Wallis test. When the overall Kruskal–Wallis test was significant, pairwise comparisons were performed using Dunn’s test with Holm adjustment for multiple comparisons. Beta-diversity differences were evaluated by PERMANOVA based on Bray–Curtis distances with 999 permutations [36]; the pseudo-F statistic, R^2^ effect size, and permutation *p* value were reported. Homogeneity of within-group multivariate dispersion was additionally evaluated using PERMDISP (betadisper) on the same Bray–Curtis distance matrix, with distances calculated to group spatial medians and significance assessed using 999 permutations [37]. Because PERMDISP detected significant heterogeneity of dispersion, the PERMANOVA result was interpreted cautiously as potentially reflecting differences in both group location and within-group dispersion. Differentially abundant taxa were identified using LEfSe [38] with an LDA score threshold > 4.0 and nominal *p* < 0.05. No FDR correction was available for the archived LEfSe output; therefore, these taxa were treated as exploratory candidate biomarkers rather than confirmatory differential-abundance findings. PICRUSt2-predicted KEGG Level 3 pathway abundances displayed in Figure 1F were compared among treatments using Kruskal–Wallis tests; the displayed *p* values were nominal and were interpreted as exploratory. For metabolomic data, unsupervised PCA was used to visualize overall sample structure and QC clustering, whereas supervised PLS-DA was used to characterize treatment-associated metabolic differences. Candidate differential metabolic features were initially screened using VIP > 1.0, FC > 1.5, or FC < 0.667, and nominal *p* < 0.05. For metabolite-level multiple-testing correction, raw *p* values for all tested metabolic features within each pairwise comparison were adjusted using the Benjamini–Hochberg FDR procedure [39], with an FDR-adjusted *p* value (q value) < 0.05 considered statistically significant. KEGG pathway-enrichment *p* values were independently adjusted using the Benjamini–Hochberg FDR procedure. Spearman rank correlations were calculated across the 15 paired rumen samples (*n* = 5 per treatment) between the six microbial taxa and 15 candidate metabolites included in Figure 2. The *p* values from all 90 microbe–metabolite comparisons were adjusted simultaneously using the Benjamini–Hochberg false discovery rate (FDR) procedure, with q < 0.05 considered significant after multiplicity correction. To evaluate the potential contribution of treatment-group separation to the pooled associations, correlation coefficients were also examined separately within each treatment as a descriptive sensitivity analysis. Because only five observations were available per treatment, these within-treatment estimates were not used for confirmatory inference. As an additional sensitivity analysis, treatment-adjusted rank correlations were obtained by rank-transforming the microbial and metabolite variables, regressing the ranks on treatment indicators, and correlating the resulting residuals. The *p* values from these 90 adjusted correlations were again subjected to Benjamini–Hochberg correction. All correlation analyses were interpreted as associative and not as evidence of direct or causal microbe–metabolite relationships. The complete pooled, treatment-specific, and treatment-adjusted correlation results are provided in Table S1.

## 3. Results

### 3.1. Growth Performance of Yaks Fed Diets with Different Roughage peNDF Levels

As shown in Table 3, no significant differences were observed among the three roughage peNDF treatments in initial body weight, final body weight, total weight gain, or average daily gain (*p* > 0.05).

### 3.2. Ruminal Volatile Fatty Acid Concentrations and Molar Proportions Under Different Roughage peNDF Levels

As shown in Table 4, the overall treatment effect on absolute butyrate concentration did not reach the predefined level of statistical significance (*p* = 0.0501), although group C had the highest numerical mean. Valerate concentration differed significantly among treatments (*p* = 0.010), with group C showing a higher value than group A, whereas group B did not differ significantly from either group. The molar proportions of isobutyrate and isovalerate also differed significantly among treatments (*p* < 0.001), with both being highest in group B, intermediate in group A, and lowest in group C. No significant treatment effects were detected for total VFA, acetate, propionate, isobutyrate, or isovalerate concentrations; for the molar proportions of acetate, propionate, butyrate, or valerate; or for the acetate-to-propionate ratio (*p* > 0.05).

### 3.3. Serum Biochemical Indices of Yaks Under Different Roughage peNDF Levels

As shown in Table 5, serum AST activity differed significantly among the dietary treatments, with a higher value observed in group A than in group B (*p* < 0.05). Serum BUN concentration was significantly higher in group B than in group C (*p* < 0.05). No significant differences were observed among the three groups in serum Ca, P, ALT, TP, ALB, CHOL, TG, ALP, GLU, or LDH levels (*p* > 0.05).

### 3.4. Rumen Microbial Community of Yaks Under Different Roughage peNDF Levels

As shown in Figure 1A, Chao1 richness differed among treatments (Kruskal–Wallis *p* = 0.0324). Dunn’s test with Holm adjustment showed that Chao1 was higher in group C than in group A (adjusted *p* = 0.0267). Shannon diversity did not differ significantly among treatments (Kruskal–Wallis *p* = 0.134). Principal coordinate analysis based on Bray–Curtis distances showed partial visual separation among the three dietary treatments. PERMANOVA detected an overall treatment-associated difference in rumen microbial community structure (pseudo-F = 1.8794, R^2^ = 0.23852, *p* = 0.002, 999 permutations; Figure 1B). However, PERMDISP also detected significant heterogeneity of within-group multivariate dispersion among treatments (F = 4.174, permutation *p* = 0.034, 999 permutations). Therefore, the PERMANOVA result cannot be attributed exclusively to differences in community centroid location and may partly reflect differences in within-group dispersion.

At the phylum level, Bacteroidota, Bacillota, Campylobacterota, and Pseudomonadota were the most abundant named phyla across the three groups (Figure 1C). At the genus level, unclassified_F082, Sulfurospirillum, Helicobacter, Rikenellaceae RC9 gut group, and Xylanibacter were among the most abundant genus-level taxa (Figure 1D).

As shown in Figure 1E, LEfSe identified exploratory candidate taxa enriched in each treatment: group A was characterized by Pseudomonadota and Escherichia–Shigella, group B by Sulfurospirillum and Lactobacillaceae, and group C by Bacteroidota, Xylanibacter, and Christensenellaceae_R-7_group. Because the LEfSe analysis used an LDA score > 4.0 and nominal *p* < 0.05 without FDR correction, these taxa are interpreted as exploratory candidate biomarkers rather than confirmatory differential-abundance findings. Figure 1F shows exploratory treatment-associated differences in the predicted relative abundances of selected KEGG Level 3 pathways, with the direction of group means varying among pathways rather than following a uniform treatment ordering. Because these profiles were inferred from 16S rRNA gene data using PICRUSt2 [29], they represent predicted functional potential rather than directly measured microbial metabolic activity.

### 3.5. Rumen Metabolic Profiles of Yaks Under Different Roughage peNDF Levels

**Figure 3:**
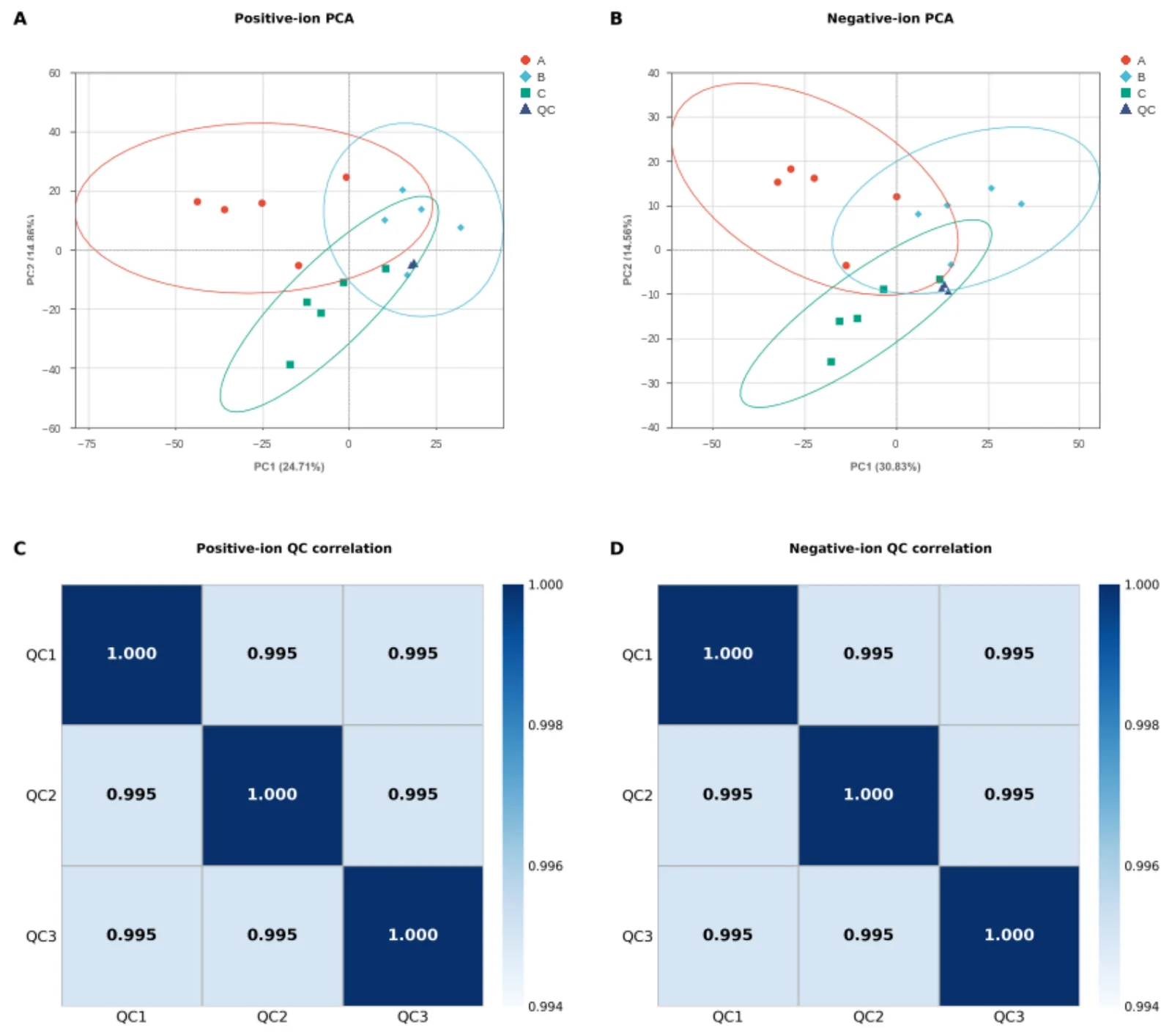
Unsupervised principal component analysis (PCA) and quality-control assessment of the rumen metabolomics dataset: (**A**) unsupervised PCA score plot of biological and pooled QC samples in positive-ion mode; (**B**) unsupervised PCA score plot of biological and pooled QC samples in negative-ion mode; (**C**) pairwise Pearson correlation matrix among pooled QC injections (QC1–QC3) in positive-ion mode; and (**D**) pairwise Pearson correlation matrix among pooled QC samples (QC1–QC3) in negative-ion mode. The pooled QC samples clustered tightly in both PCA score plots, and all pairwise Pearson correlation coefficients among QC1–QC3 were 0.995 in both ionization modes, indicating high analytical reproducibility.

Unsupervised PCA was performed to evaluate the overall distribution of the rumen metabolomic profiles and the analytical reproducibility of the dataset (Figure 3A,B). In positive-ion mode, PC1 and PC2 explained 24.71% and 14.86% of the total variance, respectively, accounting for 39.57% cumulatively. In negative-ion mode, PC1 and PC2 explained 30.83% and 14.56% of the total variance, respectively, accounting for 45.39% cumulatively. The biological samples showed partial treatment-associated separation with some overlap, whereas the pooled QC injections clustered tightly in both ionization modes. Consistently, all pairwise Pearson correlation coefficients among QC1-QC3 were 0.995 in both positive- and negative-ion modes (Figure 3C,D), indicating high analytical reproducibility. In addition, only metabolites with CV values < 30% across the pooled QC samples were retained for downstream analysis.

**Figure 4:**
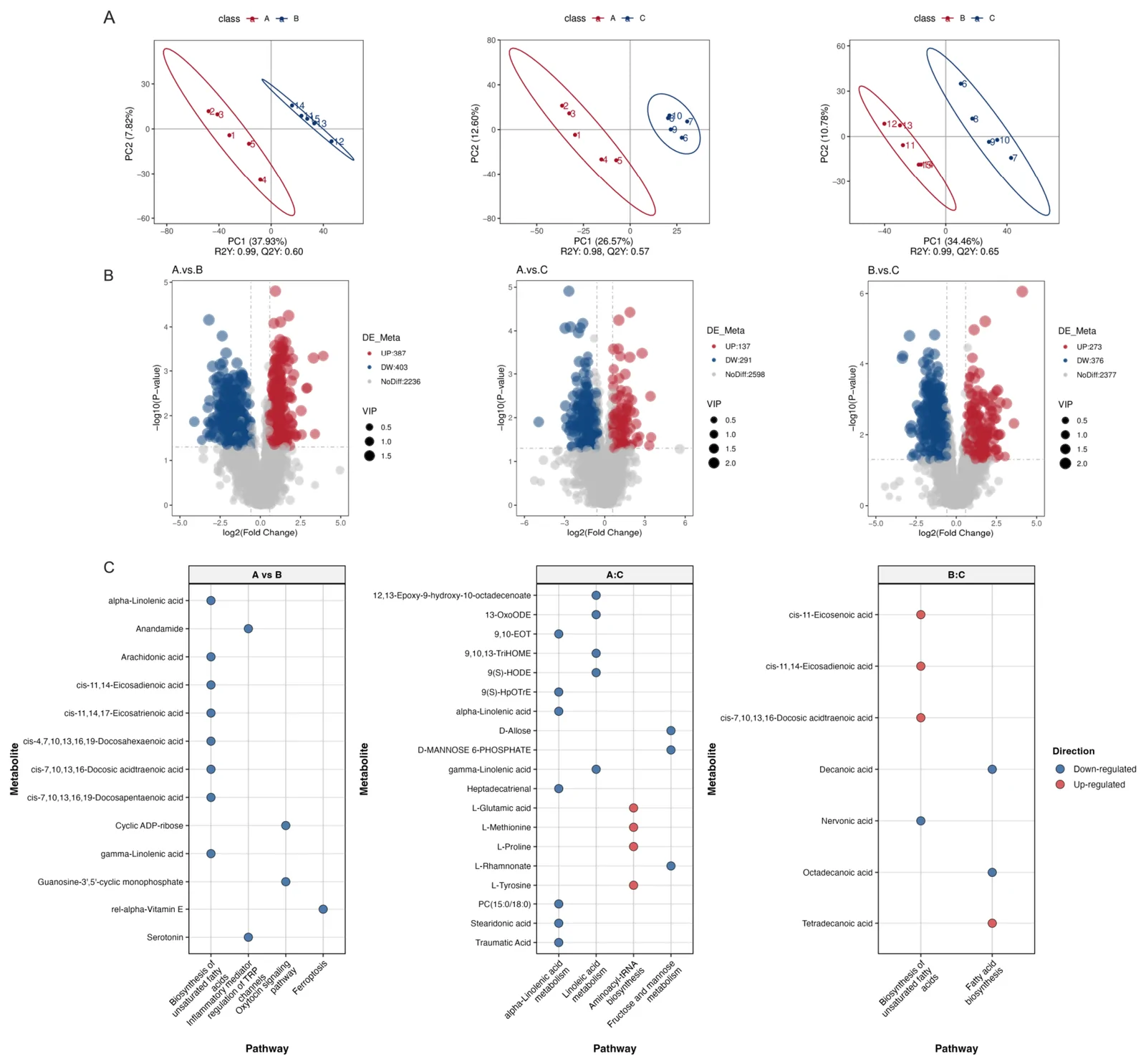
Effects of different roughage peNDF levels on rumen metabolomic profiles in yaks (*n* = 5 biological replicates per treatment): (**A**) PLS-DA; (**B**) volcano plots of candidate differential metabolic features identified using the original screening criteria of VIP > 1.0, FC > 1.5 or FC < 0.667, and nominal *p* < 0.05; (**C**) exploratory KEGG pathway-enrichment analysis. Benjamini–Hochberg FDR correction was applied to pathway-enrichment *p* values, and no pathway remained significant after correction. PLS-DA permutation testing is provided in the Supplementary Materials.

The supervised PLS-DA models further showed separation of rumen metabolic profiles among the dietary treatments (Figure 4A). Pairwise comparisons yielded R^2^Y/Q^2^Y values of 0.99/0.60 for A vs. B, 0.98/0.57 for A vs. C, and 0.99/0.65 for B vs. C. Permutation testing was performed to assess potential model overfitting, with the corresponding results provided in the Supplementary Materials.

Candidate differential metabolites were screened using the predefined criteria of VIP > 1.0, *p* < 0.05, and FC > 1.5 or FC < 0.667. Under these original screening criteria, 790 candidate differential metabolites were identified between groups A and B, including 387 upregulated and 403 downregulated metabolites (Figure 4B). In the comparison between groups A and C, 428 candidate differential metabolites were detected, including 137 upregulated and 291 downregulated metabolites. Between groups B and C, 649 candidate differential metabolites were identified, including 273 upregulated and 376 downregulated metabolites. These features were regarded as candidate differential metabolites under the original screening criteria; the complete candidate list is provided in the Supplementary Materials.

Because these features were identified using nominal, unadjusted *p* values, they were regarded as exploratory candidate differential features. After Benjamini–Hochberg FDR correction across all 3026 tested metabolic features within each pairwise comparison, 1, 8, and 95 features had FDR-adjusted *p* values (q values) < 0.05 for A vs. B, A vs. C, and B vs. C, respectively. Among these FDR-significant features, 1, 8, and 93 features, respectively, also met the original VIP > 1.0 and FC > 1.5 or FC < 0.667 criteria. The full metabolite-level output, including both raw and FDR-adjusted *p* values, is provided in the Supplementary Materials.

At the nominal, unadjusted level, KEGG pathway enrichment results were mainly related to lipid, amino acid, and carbohydrate metabolism (Figure 4C), including candidate signals involving unsaturated fatty acid biosynthesis, alpha-linolenic acid metabolism, linoleic acid metabolism, aminoacyl-tRNA biosynthesis, and fructose/mannose metabolism. However, after Benjamini–Hochberg FDR correction across the complete set of tested pathways, no pathway remained significant at adjusted *p* < 0.05. Accordingly, these pathway-level findings are treated as exploratory, hypothesis-generating signals rather than evidence of confirmed pathway activation or a demonstrated mechanism.

### 3.6. Associations Between Selected Rumen Microbial Taxa and Candidate Metabolites

Spearman rank-correlation analysis was conducted across the 15 paired rumen samples (*n* = 5 per treatment) for the six microbial taxa and 15 candidate metabolites included in Figure 2. After Benjamini–Hochberg correction for the 90 tested microbe–metabolite pairs, nine pooled correlations remained significant at q < 0.05. Christensenellaceae_R-7_group was positively correlated with D-mannose 6-phosphate (ρ = 0.811, q = 0.015), all-rac-α-tocopherol (ρ = 0.736, q = 0.032), and α-linolenic acid (ρ = 0.686, q = 0.048). Escherichia–Shigella was negatively correlated with α-linolenic acid (ρ = −0.789, q = 0.015) and all-rac-α-tocopherol (ρ = −0.764, q = 0.020). Sulfurospirillum showed positive correlations with all-cis-7,10,13,16-docosatetraenoic acid (ρ = 0.786, q = 0.015), tetradecanoic acid (ρ = 0.718, q = 0.034), and heptadecatrienal (ρ = 0.711, q = 0.034), whereas Prevotella was negatively correlated with tetradecanoic acid (ρ = −0.715, q = 0.034).

To assess the potential influence of treatment-group separation on these pooled associations, correlation coefficients were also examined separately within treatments as a descriptive sensitivity analysis (Table S1). Their direction and magnitude were not consistently preserved across all three groups. Because each treatment contained only five biological replicates, the within-treatment coefficients were not subjected to confirmatory inference. For Prevotella, the within-group coefficient could not be estimated in group B because its abundance showed no variation within that treatment. In the treatment-adjusted rank-correlation analysis, none of the 90 associations remained significant after FDR correction (all q > 0.05). Thus, the pooled correlations may partly reflect treatment-associated between-group separation and should not be interpreted as evidence of treatment-independent microbe–metabolite coupling.

## 4. Discussion

Physically effective neutral detergent fiber (peNDF) integrates dietary fiber concentration with feed particle structure and is an important descriptor of the physical effectiveness of ruminant diets [8,9]. In the present study, three reported as-fed roughage peNDF_8.00_ treatment labels (11.52%, 13.16%, and 15.43%) were evaluated in housed yaks. Dietary treatment did not significantly affect final body weight, total weight gain, or average daily gain. Group C had the highest numerical absolute butyrate concentration (*p* = 0.0501) and a higher valerate concentration than group A, while the molar proportions of isobutyrate and isovalerate also differed among treatments; group C showed higher Chao1 richness than group A, whereas Shannon diversity did not differ significantly among treatments, and LEfSe identified exploratory treatment-associated candidate taxa. Untargeted metabolomics also identified numerous candidate differential features, but no tested KEGG pathway remained significant after Benjamini–Hochberg FDR correction. Accordingly, the results support treatment-associated differences in rumen fermentation and metabolomic profiles, together with variation in microbial community structure, rather than an established optimal peNDF requirement or demonstrated improvement in rumen stability.

Growth responses to peNDF are not uniform across ruminant species and diets. Wang et al. [40] found that dry matter intake and average daily gain of Holstein replacement heifers were not significantly affected across dietary peNDF levels, although chewing behavior, digestibility, and rumen fermentation changed. Jang et al. [41] similarly reported that altering peNDF in Korean native goats affected physical and digestive responses without consistent changes in dry matter intake or average daily gain. By contrast, Godínez-Olmedo et al. [42] observed a nonlinear growth response in finishing lambs, with performance greatest around an intermediate peNDF level. Together, these studies indicate that growth responses depend on species, diet, and the peNDF range evaluated. Consistent with the heifer and goat studies, the present yak experiment showed no significant differences in final body weight, total weight gain, or average daily gain among treatments.

Rumen fermentation responses were selective rather than uniform across the measured VFA variables. The overall treatment effect on absolute butyrate concentration narrowly missed the predefined threshold for statistical significance (*p* = 0.0501), although group C had the highest numerical mean. Valerate concentration was significantly higher in group C than in group A, whereas group B did not differ significantly from either group. The molar proportions of isobutyrate and isovalerate also differed among treatments, whereas the molar proportions of acetate, propionate, butyrate, and valerate, as well as the acetate-to-propionate ratio, were not significantly affected by dietary treatment. Collectively, these findings indicate selective changes in individual fermentation products rather than a consistent shift in the major ruminal VFA fermentation pattern across the roughage peNDF treatments.

VFAs are major energy substrates in ruminants, and butyrate has established trophic and metabolic roles in the ruminal epithelium [43,44]. The numerical increase in absolute butyrate concentration in group C nevertheless warrants consideration because it differs from fermentation patterns commonly discussed in relation to increasing dietary physical effectiveness [8,45]. In the present study, roughage physical effectiveness was manipulated by altering oat-hay particle size while maintaining the same ingredient composition and inclusion rates, rather than by increasing forage inclusion. Therefore, the fermentation response observed here may not necessarily parallel that associated with changes in forage proportion. Alterations in particle size may affect chewing activity, ruminal mat characteristics, and digesta passage [46], and may subsequently influence substrate availability to rumen microorganisms. However, these processes were not directly measured in the present study and should therefore be regarded as possible explanations rather than demonstrated mechanisms. Moreover, the measured ruminal VFA concentrations reflect the net balance among production, absorption, dilution, and outflow [47,48]. Accordingly, the numerical increase in absolute butyrate concentration should not be interpreted as evidence of increased butyrate production or improved energy utilization.

Most serum biochemical indices were unaffected by dietary treatment. Serum AST activity differed among treatments (overall *p* = 0.016), with higher activity in group A than in group B, whereas group C did not differ significantly from either group. Because AST is not liver-specific and may originate from both hepatic and skeletal muscle tissues [20], this isolated difference should be interpreted cautiously and cannot, by itself, be taken as evidence of altered hepatic function. Serum ALT activity was not significantly affected by treatment, further supporting a conservative interpretation of the AST response. Serum BUN concentration was higher in group B than in group C. Because BUN reflects whole-body nitrogen metabolism and has been associated with nitrogen excretion and nitrogen-use efficiency [49,50], this difference should not be interpreted as direct evidence of improved nitrogen utilization. In the absence of measurements of ruminal NH_3_-N, microbial protein synthesis, urinary nitrogen excretion, and nitrogen balance, the biological significance of the BUN response remains uncertain.

Changes in the rumen microbial community suggested treatment-associated variation beyond the measured fermentation end-products. Group C showed higher Chao1 richness than group A, whereas Shannon diversity did not differ significantly among treatments; PCoA displayed partial visual separation among treatments. PERMANOVA detected an overall treatment-associated difference in Bray–Curtis community structure; however, PERMDISP also revealed significant heterogeneity of within-group multivariate dispersion. Because PERMANOVA may be influenced by differences in dispersion as well as group location, the observed beta-diversity pattern cannot be attributed solely to a shift in community centroids [37,51]. Accordingly, these results are more appropriately interpreted as evidence of treatment-associated variation in rumen microbial community structure rather than complete or unequivocal separation of microbial communities among treatments. Rumen microbial communities are known to respond to diet and host factors [52,53], and dietary physical structure can alter chewing behavior and the ruminal environment [54,55]. Nevertheless, ruminal pH and chewing/rumination behavior were not measured, and the small microbiome sample size (*n* = 5 per treatment) further limits the precision and generalizability of the community-level inference.

The taxonomic shifts observed in the present study provide additional, but exploratory, context. LEfSe identified Pseudomonadota and Escherichia–Shigella as candidate biomarkers of group A, Sulfurospirillum and Lactobacillaceae as candidate biomarkers of group B, and Bacteroidota, Xylanibacter, and Christensenellaceae_R-7_group as candidate biomarkers of group C. Prevotella was not identified as an LEfSe biomarker in the rechecked analysis but was retained among the taxa selected for the cross-omic correlation analysis; members of Prevotella are metabolically diverse and include species capable of using polysaccharides and proteins in the rumen [56,57]. Christensenellaceae_R-7_group is a common yak-rumen taxon and has been associated with diet-dependent forage-to-concentrate conditions in yak studies [58]. These LEfSe signals and cross-omic associations are exploratory and do not establish direct functional contributions to the observed fermentation products. Pseudomonadota, historically referred to as Proteobacteria, have been proposed as dysbiosis-associated taxa in other gut ecosystems [59], and high-concentrate feeding can alter rumen microbial composition in cattle [60]; however, enrichment of Pseudomonadota and Escherichia–Shigella in group A is not direct evidence of rumen dysbiosis or reduced microbial stability because rumen health and stability were not directly measured.

PICRUSt2-based functional prediction suggested treatment-associated differences in predicted pathways related to carbon metabolism, amino acid biosynthesis, cofactor biosynthesis, microbial metabolism in diverse environments, secondary-metabolite biosynthesis, and overall metabolism. The displayed group means varied by pathway rather than showing a uniform monotonic response: group C had the highest predicted means for carbon metabolism, amino acid biosynthesis, secondary-metabolite biosynthesis, and metabolic pathways; group B had the highest mean for cofactor biosynthesis; and group A had the highest mean for microbial metabolism in diverse environments. Because PICRUSt2 infers functional potential from marker-gene data rather than directly measuring genes or transcripts, these results should not be interpreted as confirmed metabolic activity [29]. Shotgun metagenomics, metatranscriptomics, or direct enzyme/activity measurements would be required to validate these predictions.

Rumen metabolomics provided an additional descriptive layer for evaluating treatment-associated metabolic variation. Under the original VIP > 1.0, FC > 1.5 or FC < 0.667, and nominal *p* < 0.05 screening criteria, numerous candidate treatment-associated metabolic features were identified. However, metabolite-level Benjamini–Hochberg FDR correction substantially reduced the number of statistically significant features, with 1, 8, and 95 features remaining at q < 0.05 for A vs. B, A vs. C, and B vs. C, respectively. Therefore, the larger sets identified using the original nominal-P screening criteria are interpreted primarily as exploratory candidate features. Nominal pathway-enrichment signals included alpha-linolenic acid metabolism, linoleic acid metabolism, aminoacyl-tRNA biosynthesis, and fructose/mannose metabolism. However, none of the tested pathway-enrichment signals remained significant after Benjamini–Hochberg FDR correction; accordingly, these pathways are discussed only as exploratory, hypothesis-generating signals. Candidate treatment-associated features traceable to the reported results included alpha-linolenic acid, stearidonic acid, gamma-linolenic acid, 9(S)-HODE, 13-OxoODE, D-allose, D-mannose 6-phosphate, and L-rhamnonate. These observations require confirmation in larger samples and by targeted metabolomics or functional validation.

The amino acid-related candidate metabolite profile also showed treatment-associated differences. Compared with group C, group A had higher levels of L-glutamic acid, L-methionine, L-proline, and L-tyrosine.

Several pooled microbe–metabolite associations remained significant after FDR correction, but the treatment structure of the dataset complicates their biological interpretation. Adjustment for study covariates is important in cross-omic correlation analysis because unaccounted confounding can create or amplify apparent microbe–metabolite associations [61]. In the present data, treatment-specific coefficients were not uniformly consistent across groups, and none of the treatment-adjusted associations remained significant after FDR correction. The attenuation of these signals after treatment adjustment therefore suggests that dietary treatment contributed to the pooled correlation pattern. In addition, correlation estimates based on microbiome relative-abundance data can be sensitive to sparsity, compositional constraints, heterogeneity, and the large-p/small-n setting, all of which can reduce stability and complicate statistical inference [62,63]. Accordingly, the pooled signals are best viewed as hypothesis-generating associations that may combine between-treatment separation with within-treatment covariation rather than as evidence of treatment-independent or causal biological coupling. Confirmation will require larger within-treatment sample sizes and targeted functional validation.

Overall, the present study indicates treatment-associated differences in rumen fermentation products and metabolomic profiles, together with variation in rumen microbial community structure and predicted microbial functional potential, while no significant treatment effect on short-term growth performance was detected. The 15.43% as-fed roughage peNDF_8.00_ treatment label was associated with the highest numerical absolute butyrate concentration (*p* = 0.0501), a higher valerate concentration than the 11.52% roughage peNDF treatment, higher Chao1 richness, and exploratory candidate enrichment of Bacteroidota, Xylanibacter, and Christensenellaceae_R-7_group. PICRUSt2-predicted pathway means varied by pathway and did not show a uniform monotonic pattern across the three treatments. These findings do not establish direct causality or an optimal peNDF requirement. Future studies using direct rumen-function measurements, individual feed-intake measurements, and larger biological sample sizes are required to clarify the underlying mechanisms.

Study limitations should be considered when interpreting these findings. The yaks were group-fed, so individual dry matter intake, refusals, feed sorting, and feed conversion ratio were not available. Ruminal pH and chewing/rumination behavior were not measured. Rumen fluid was collected once before morning feeding using an oral sampling tube, which can introduce salivary contamination and does not characterize diurnal variation. The 10-day adaptation period was relatively short. The microbiome and metabolomics analyses were based on only *n* = 5 biological replicates per treatment; a separately sequenced negative-control library was unavailable, and significant heterogeneity of multivariate dispersion was detected in the Bray–Curtis beta-diversity analysis. Consequently, the PERMANOVA result cannot be interpreted solely as evidence of differences in community centroid location. The small within-treatment sample size also limited the stability of treatment-specific microbe–metabolite correlations, while pooled correlations could not fully distinguish within-treatment covariation from treatment-associated group separation.

## 5. Conclusions

This study showed that, within the present 60-day experimental period, the three as-fed roughage peNDF_8.00_ treatments were associated with differences in rumen fermentation and rumen metabolomic profiles, together with variation in rumen microbial community structure, while no significant treatment effect on short-term growth performance was detected. Compared with the lower roughage peNDF_8.00_ treatments, the 15.43% roughage peNDF treatment showed the highest numerical absolute ruminal butyrate concentration (*p* = 0.0501), a higher valerate concentration than the 11.52% roughage peNDF treatment, higher Chao1 richness, and exploratory candidate enrichment of Bacteroidota, Xylanibacter, and Christensenellaceae_R-7_group. These treatment-associated patterns should not be interpreted as proof of improved rumen stability or as an established optimal peNDF requirement. Longer-term studies with individual feed-intake measurements, nutrient digestibility, direct rumen-function measurements, and larger omics sample sizes are needed.

## Figures and Tables

**Table 1:** Ingredient composition (%) and chemical composition of the experimental diet.

Items			
Ingredients			
Corn silage	23.77	Linseed cake	3.64
Green hay	8.12	NaHCO_3_	0.86
Stalk	13.64	NaCl	0.62
Corn grain	42.99	Limestone powder (CaCO_3_)	0.71
Cottonseed meal	4.65	Premix1	1.00
Total	100		
Chemical composition of the experimental diets			
Moisture, % as-fed	58.80	EE, % DM	2.43
DM, % as-fed	41.20	Roughage NDF^2^ (% DM)	42.31
CP, % DM	8.74	ADF, % DM	21.60
Ash, % DM	3.40	ME, MJ/kg DM	9.80

Note: The premix provided the following per kilogram of diet: vitamin A, 10,000 IU; vitamin D_3_, 2000 IU; vitamin E, 30 mg; Fe, 50 mg; Cu, 10 mg; Zn, 50 mg; Mn, 40 mg; I, 0.5 mg; Se, 0.3 mg; and Co, 0.2 mg. DM, dry matter; CP, crude protein; EE, ether extract; NDF, neutral detergent fiber; ADF, acid detergent fiber. DM and moisture are reported on an as-fed basis; CP, EE, NDF, ash, and ADF are reported on a dry-matter basis. Corn silage refers to fermented whole-plant corn, whereas corn grain refers to the cereal grain component. The ingredient previously labeled as “talcum powder” was feed-grade limestone powder (CaCO_3_), included as a mineral calcium source. ME, metabolizable energy; the reported ME value (9.80 MJ/kg DM) was used for dietary energy evaluation with reference to NASEM (2016) [15]. The NDF value used for peNDF_8.00_ calculation was determined in the roughage mixture subjected to particle-size separation and does not represent the NDF concentration of the complete diet including the separately supplied concentrate.

**Table 2:** Dry matter retained on each sieve and roughage peNDF_8.00_ values of the experimental treatments.

Items	Group		
	A	B	C
DM retained (%)			
>19.00 mm	46.32	54.48	65.34
8.00–19.00 mm	19.77	21.02	23.19
4.00–8.00 mm	17.39	14.66	6.43
≤4.00 mm	16.52	9.84	5.04
pef (%) ①			
pef_8.00_	66.09	75.50	88.53
Roughage peNDF contents (%) ②			
Roughage peNDF_8.00_	11.52	13.16	15.43

① pef_8.00_ represents the proportion of dry matter retained on the >19.00 mm and 8.00–19.00 mm sieves relative to total dry matter. ② Roughage peNDF_8.00_ (% as-fed) = roughage NDF (% as-fed; 17.43%) × roughage pef_8.00_. The separately supplied concentrate was not included in either the particle-size separation or the roughage peNDF_8.00_ calculation.

**Table 3:** Effects of different roughage peNDF levels on growth performance of yaks.

Items	Groups			SEM	*p*-Value
	A	B	C		
IBW/kg	238.30	241.40	235.50	12.48	0.946
FBW/kg	273.90	277.90	279.20	12.82	0.955
TWG/kg	35.60	36.50	43.70	3.07	0.143
ADG/(g/d)	593.33	608.33	728.33	51.16	0.143

Note: IBW, initial body weight; FBW, final body weight; TWG, total weight gain; ADG, average daily gain; SEM, standard error of the mean. Growth-performance analyses were based on *n* = 10 yaks per treatment.

**Table 4:** Effects of different roughage peNDF levels on ruminal volatile fatty acid concentrations, molar proportions, and the acetate-to-propionate ratio in yaks (*n* = 5 biological replicates per treatment).

Items	Groups			SEM	*p*-Value
	A	B	C		
	Concentration, mmol/L				
Total VFA	52.346	51.052	67.113	5.329	0.249
Acetate	36.871	35.248	46.215	3.656	0.289
Propionate	8.765	8.706	11.902	1.012	0.174
Butyrate	4.724	4.819	6.712	0.569	0.0501
Isobutyrate	0.742	0.832	0.765	0.072	0.668
Valerate	0.650 ^b^	0.726 ^ab^	0.921 ^a^	0.053	0.010
Isovalerate	0.593	0.721	0.598	0.057	0.246
	Molar proportion, %				
Acetate	70.454	69.215	68.828	0.516	0.107
Propionate	16.695	16.932	17.733	0.357	0.141
Butyrate	9.022	9.380	10.001	0.355	0.185
Isobutyrate	1.423 ^b^	1.629 ^a^	1.149 ^c^	0.050	<0.001
Valerate	1.263	1.433	1.391	0.066	0.206
Isovalerate	1.143 ^b^	1.411 ^a^	0.899 ^c^	0.040	<0.001
Acetate/propionate	4.231	4.104	3.883	0.116	0.141

Note: SEM, pooled standard error of the treatment mean. Molar proportions were calculated separately for each biological sample as the concentration of each individual VFA divided by the sum of the concentrations of the six quantified VFAs × 100. Different superscript lowercase letters within the same row indicate significant pairwise differences among treatments (adjusted *p* < 0.05). Tukey’s HSD test was used for pairwise comparisons following a significant one-way ANOVA, whereas Dunn’s test with Holm adjustment was used following a significant Kruskal–Wallis test, as appropriate.

**Table 5:** Effects of different roughage peNDF levels on serum biochemical indices of yaks (*n* = 10 per treatment).

Items	Groups			SEM	*p*-Value
	A	B	C		
Ca (mmol/L)	1.98	2.00	2.01	0.02	0.716
P (mmol/L)	2.11	2.35	2.24	0.07	0.428
ALT/(U/L)	34.42	35.30	45.66	2.37	0.095
AST/(U/L)	115.51 ^a^	93.18 ^b^	104.10 ^ab^	3.30	0.016
TP(g/L)	71.25	70.12	80.89	2.46	0.145
ALB/(g/L)	27.00	27.95	28.99	0.74	0.563
CHOL/(mmol/L)	2.54	2.48	2.95	0.11	0.224
TG/(mmol/L)	0.34	0.28	0.45	0.04	0.153
ALP/(U/L)	182.86	164.65	166.84	8.51	0.652
BUN(mmol/L)	4.57 ^ab^	5.06 ^a^	4.11 ^b^	0.23	0.013
GLU/(mmol/L)	5.05	4.88	5.13	0.09	0.495
LDH/(U/L)	1181.73	1051.97	1196.37	36.64	0.214

Note: Different superscript lowercase letters within the same row indicate significant pairwise differences among treatments (adjusted *p* < 0.05). Tukey’s HSD test was used for pairwise comparisons following a significant one-way ANOVA, whereas Dunn’s test with Holm adjustment was used following a significant Kruskal–Wallis test, as appropriate.

## Data Availability

The 16S rRNA gene sequencing data generated in this study have been deposited in the NCBI Sequence Read Archive (SRA) under accession number PRJNA1498289.

## Supplementary Materials

The following supporting information can be downloaded at: https://www.mdpi.com/article/10.3390/microorganisms14102298/s1, Table S1. Rarefaction curves of the rumen microbial communities, treatment-specific, and treatment-adjusted Spearman correlations between selected rumen microbial taxa and candidate metabolites.

## Abbreviations

The following abbreviations are used in this manuscript:

ASV	Amplicon Sequence Variant
ANOVA	Analysis of Variance
PCA	Principal Component Analysis
PCoA	Principal Coordinate Analysis
LEfSe	Linear Discriminant Analysis Effect Size
PLS-DA	Partial Least Squares Discriminant Analysis
VIP	Variable Importance in Projection
peNDF	Physically Effective Neutral Detergent Fiber
DM	Dry Matter
CP	Crude Protein
EE	Ether Extract
NDF	Neutral Detergent Fiber
ADF	Acid Detergent Fiber
VFA	Volatile Fatty Acid
TVFA	Total Volatile Fatty Acid
BUN	Blood Urea Nitrogen
DMI	Dry Matter Intake
PSPS	Penn State Particle Separator
PICRUSt2	Phylogenetic Investigation of Communities by Reconstruction of Unobserved States 2
KEGG	Kyoto Encyclopedia of Genes and Genomes
HMDB	Human Metabolome Database
