# Supplementary material for: Effects of Roughage Physically Effective Neutral Detergent Fiber Levels on Rumen Fermentation, Microbiota, and Metabolomic Profiles in Yaks
Source: Microorganisms. 2026 Oct 9;14(10):2298. doi: 10.3390/microorganisms14102298 (PMC13707538; doi:10.3390/microorganisms14102298)
Supplement: Supplementary file 1 [file microorganisms-14-02298-s001.zip › microorganisms-4497004-supplementary.pdf]

Table S1. Rarefaction curves of the rumen microbial communities, treatment-specific, and treatment-adjusted Spearman correlations between selected rumen microbial taxa and candidate metabolites.

Microbial taxon	Metabolite	Pooled ρ	Pooled P	FDR q	A ρ	B ρ	C ρ	Treatment-adjusted ρ	Adjusted P	Adjusted FDR q
Christensenellaceae R-7 group	all-rac-α-Tocopherol	**0.736**	0.002	**0.032**	-0.300	0.700	0.400	0.350	0.241	0.805
Christensenellaceae R-7 group	α-Linolenic acid	**0.686**	0.005	**0.048**	0.100	0.500	0.700	0.330	0.271	0.805
Christensenellaceae R-7 group	PC(15:0/18:0)	0.664	0.007	0.062	-0.300	0.300	0.800	0.117	0.704	0.941
Christensenellaceae R-7 group	D-Mannose 6-phosphate	**0.811**	<0.001	**0.015**	0.600	0.000	0.700	0.170	0.578	0.885
Christensenellaceae R-7 group	L-Proline	-0.454	0.089	0.252	0.000	0.500	-0.100	0.127	0.678	0.939
Christensenellaceae R-7 group	Heptadecatrienal	0.411	0.128	0.269	0.200	-0.700	-0.600	-0.446	0.127	0.601
Christensenellaceae R-7 group	12,13-Epoxy-9-hydroxy-10-octadecenoate	0.611	0.016	0.108	0.100	-0.500	-0.700	-0.500	0.082	0.474
Christensenellaceae R-7 group	all-cis-7,10,13,16-Docosatetraenoic acid	0.204	0.467	0.627	0.000	0.000	-0.500	-0.230	0.449	0.813
Christensenellaceae R-7 group	Cyclic ADP-ribose	0.371	0.173	0.307	-1.000	-0.700	-0.400	-0.592	0.033	0.459
Christensenellaceae R-7 group	9,10-EOT	0.543	0.037	0.157	-0.100	-0.900	-0.300	-0.501	0.081	0.474
Christensenellaceae R-7 group	9(S)-HpOTrE	0.507	0.054	0.186	0.100	-0.700	-0.200	-0.496	0.084	0.474
Christensenellaceae R-7 group	Tetradecanoic acid	-0.182	0.516	0.663	-0.300	-0.100	0.900	-0.110	0.720	0.941
Christensenellaceae R-7 group	γ-Linolenic acid	0.479	0.071	0.229	0.200	0.100	-0.800	-0.291	0.335	0.805
Christensenellaceae R-7 group	13-OxoODE	0.471	0.076	0.236	-0.100	-0.800	-0.700	-0.621	0.023	0.459
Christensenellaceae R-7 group	cis-11,14-Eicosadienoic acid	0.104	0.713	0.813	0.300	0.300	0.100	0.140	0.648	0.925
Escherichia-Shigella	all-rac-α-Tocopherol	**-0.764**	<0.001	**0.020**	-0.800	-0.900	-0.100	-0.542	0.056	0.474
Escherichia-Shigella	α-Linolenic acid	**-0.789**	<0.001	**0.015**	-0.600	-0.800	-0.500	-0.512	0.074	0.474
Escherichia-Shigella	PC(15:0/18:0)	-0.375	0.168	0.307	0.700	-0.600	-0.300	-0.110	0.721	0.941
Escherichia-Shigella	D-Mannose 6-phosphate	-0.568	0.027	0.153	-0.400	-0.200	-0.300	-0.168	0.584	0.885
Escherichia-Shigella	L-Proline	-0.068	0.810	0.848	0.100	-0.800	0.600	0.026	0.934	0.967
Escherichia-Shigella	Heptadecatrienal	-0.543	0.037	0.157	-0.300	0.600	0.600	0.379	0.201	0.777
Escherichia-Shigella	12,13-Epoxy-9-hydroxy-10-octadecenoate	-0.429	0.111	0.263	-0.100	0.600	0.700	0.585	0.036	0.459
Escherichia-Shigella	all-cis-7,10,13,16-Docosatetraenoic acid	-0.421	0.118	0.265	-0.100	-0.100	0.900	0.392	0.185	0.757
Escherichia-Shigella	Cyclic ADP-ribose	-0.393	0.147	0.289	0.300	0.600	0.600	0.410	0.165	0.705
Escherichia-Shigella	9,10-EOT	-0.468	0.079	0.236	-0.600	0.700	0.300	0.263	0.385	0.805
Escherichia-Shigella	9(S)-HpOTrE	-0.521	0.046	0.181	-0.100	0.600	0.200	0.341	0.254	0.805
Escherichia-Shigella	Tetradecanoic acid	-0.389	0.152	0.290	-0.300	0.500	-0.500	-0.201	0.511	0.821
Escherichia-Shigella	γ-Linolenic acid	-0.443	0.098	0.253	-0.300	-0.200	1.000	0.490	0.090	0.474
Escherichia-Shigella	13-OxoODE	-0.425	0.114	0.264	-0.500	0.600	0.700	0.491	0.088	0.474
Escherichia-Shigella	cis-11,14-Eicosadienoic acid	-0.368	0.177	0.307	-0.700	0.100	0.100	0.053	0.862	0.966
Prevotella	all-rac-α-Tocopherol	-0.211	0.451	0.627	-0.224	NA	-0.671	-0.212	0.487	0.813
Prevotella	α-Linolenic acid	-0.298	0.281	0.443	-0.112	NA	-0.894	-0.270	0.373	0.805
Prevotella	PC(15:0/18:0)	-0.087	0.758	0.832	-0.224	NA	-0.671	-0.265	0.382	0.805
Prevotella	D-Mannose 6-phosphate	-0.087	0.758	0.832	-0.447	NA	-0.335	-0.212	0.488	0.813
Prevotella	L-Proline	-0.160	0.568	0.710	0.783	NA	0.224	0.375	0.207	0.777
Prevotella	Heptadecatrienal	-0.206	0.461	0.627	-0.783	NA	0.783	0.085	0.783	0.957
Prevotella	12,13-Epoxy-9-hydroxy-10-octadecenoate	-0.069	0.808	0.848	-0.783	NA	0.447	0.057	0.854	0.966
Prevotella	all-cis-7,10,13,16-Docosatetraenoic acid	-0.316	0.251	0.403	-0.783	NA	0.447	-0.025	0.935	0.967
Prevotella	Cyclic ADP-ribose	-0.156	0.579	0.714	-0.447	NA	0.335	-0.007	0.981	0.991
Prevotella	9,10-EOT	-0.197	0.481	0.637	-0.671	NA	-0.112	-0.202	0.508	0.821
Prevotella	9(S)-HpOTrE	-0.206	0.461	0.627	-0.783	NA	0.112	-0.079	0.797	0.957
Prevotella	Tetradecanoic acid	**-0.715**	0.003	**0.034**	-0.894	NA	-0.671	-0.697	0.008	0.459
Prevotella	γ-Linolenic acid	-0.069	0.808	0.848	-0.783	NA	0.783	0.222	0.466	0.813
Prevotella	13-OxoODE	-0.119	0.672	0.786	-0.894	NA	0.447	0.033	0.915	0.967
Prevotella	cis-11,14-Eicosadienoic acid	-0.128	0.649	0.768	0.224	NA	-0.224	0.216	0.478	0.813
p-251-o5	all-rac-α-Tocopherol	0.486	0.066	0.221	0.500	-0.500	-0.600	-0.082	0.789	0.957
p-251-o5	α-Linolenic acid	0.446	0.095	0.253	0.900	-0.600	-0.300	-0.100	0.745	0.957
p-251-o5	PC(15:0/18:0)	0.582	0.023	0.147	0.000	0.000	0.200	0.156	0.611	0.902
p-251-o5	D-Mannose 6-phosphate	0.650	0.009	0.071	0.600	-0.900	0.700	0.062	0.839	0.966
p-251-o5	L-Proline	-0.393	0.147	0.289	-0.100	-0.600	0.100	-0.065	0.834	0.966
p-251-o5	Heptadecatrienal	0.411	0.128	0.269	0.500	-0.300	0.100	-0.043	0.888	0.966
p-251-o5	12,13-Epoxy-9-hydroxy-10-octadecenoate	0.507	0.054	0.186	0.600	-0.300	-0.800	-0.233	0.444	0.813
p-251-o5	all-cis-7,10,13,16-Docosatetraenoic acid	0.232	0.405	0.598	0.100	-0.200	-0.400	0.012	0.968	0.990
p-251-o5	Cyclic ADP-ribose	0.368	0.177	0.307	-0.300	-0.300	-0.100	-0.171	0.577	0.885
p-251-o5	9,10-EOT	0.429	0.111	0.263	0.900	-0.400	-1.000	-0.290	0.337	0.805
p-251-o5	9(S)-HpOTrE	0.461	0.084	0.244	0.600	-0.300	-0.500	-0.144	0.640	0.925

Microbial taxon	Metabolite	Pooled ρ	Pooled P	FDR q	A ρ	B ρ	C ρ	Treatment-adjusted ρ	Adjusted P	Adjusted FDR q
p-251-o5	Tetradecanoic acid	-0.004	0.990	0.990	0.200	0.400	0.400	0.257	0.396	0.810
p-251-o5	γ-Linolenic acid	0.443	0.098	0.253	0.500	-0.500	-0.300	-0.038	0.902	0.967
p-251-o5	13-OxoODE	0.396	0.143	0.289	0.600	-0.700	-0.800	-0.295	0.328	0.805
p-251-o5	cis-11,14-Eicosadienoic acid	-0.104	0.713	0.813	0.000	0.300	-0.600	-0.328	0.273	0.805
Lactobacillus	all-rac-α-Tocopherol	-0.036	0.899	0.910	-0.100	0.000	-0.100	0.271	0.370	0.805
Lactobacillus	α-Linolenic acid	0.050	0.860	0.879	0.000	0.100	-0.300	0.298	0.323	0.805
Lactobacillus	PC(15:0/18:0)	-0.521	0.046	0.181	-0.600	-0.500	-0.700	-0.307	0.308	0.805
Lactobacillus	D-Mannose 6-phosphate	-0.361	0.187	0.311	0.300	-0.100	-0.800	-0.057	0.852	0.966
Lactobacillus	L-Proline	0.557	0.031	0.155	0.200	0.100	-0.600	0.091	0.767	0.957
Lactobacillus	Heptadecatrienal	-0.096	0.732	0.824	-0.100	-0.700	0.400	-0.320	0.287	0.805
Lactobacillus	12,13-Epoxy-9-hydroxy-10-octadecenoate	-0.204	0.467	0.627	-0.300	-0.200	0.800	-0.003	0.991	0.991
Lactobacillus	all-cis-7,10,13,16-Docosatetraenoic acid	0.068	0.810	0.848	-0.200	-0.800	0.000	-0.275	0.364	0.805
Lactobacillus	Cyclic ADP-ribose	-0.429	0.111	0.263	-0.900	-0.700	-0.400	-0.641	0.018	0.459
Lactobacillus	9,10-EOT	-0.279	0.315	0.480	-0.300	-0.600	0.700	-0.165	0.590	0.885
Lactobacillus	9(S)-HpOTrE	-0.221	0.428	0.621	-0.300	-0.700	0.800	-0.281	0.352	0.805
Lactobacillus	Tetradecanoic acid	0.282	0.308	0.478	-0.400	0.600	-0.600	-0.247	0.416	0.813
Lactobacillus	γ-Linolenic acid	-0.057	0.840	0.869	-0.100	-0.500	0.200	-0.049	0.873	0.966
Lactobacillus	13-OxoODE	-0.164	0.558	0.708	-0.300	-0.300	0.800	-0.087	0.778	0.957
Lactobacillus	cis-11,14-Eicosadienoic acid	0.511	0.052	0.186	0.600	0.700	0.600	0.422	0.151	0.680
Sulfurospirillum	all-rac-α-Tocopherol	0.182	0.516	0.663	-0.300	-0.300	0.000	-0.132	0.667	0.939
Sulfurospirillum	α-Linolenic acid	0.236	0.398	0.597	-0.600	-0.100	-0.100	-0.247	0.416	0.813
Sulfurospirillum	PC(15:0/18:0)	-0.129	0.648	0.768	-0.300	0.300	-0.400	-0.120	0.697	0.941
Sulfurospirillum	D-Mannose 6-phosphate	0.139	0.621	0.755	0.600	0.100	-0.500	0.215	0.481	0.813
Sulfurospirillum	L-Proline	0.361	0.187	0.311	-0.900	-0.100	-0.700	-0.635	0.020	0.459
Sulfurospirillum	Heptadecatrienal	**0.711**	0.003	**0.034**	0.700	0.700	0.300	0.550	0.051	0.474
Sulfurospirillum	12,13-Epoxy-9-hydroxy-10-octadecenoate	0.386	0.156	0.292	0.400	0.200	0.600	0.348	0.243	0.805
Sulfurospirillum	all-cis-7,10,13,16-Docosatetraenoic acid	**0.786**	<0.001	**0.015**	0.900	0.300	-0.200	0.493	0.087	0.474
Sulfurospirillum	Cyclic ADP-ribose	0.336	0.221	0.362	-0.200	0.700	-0.700	-0.042	0.891	0.966
Sulfurospirillum	9,10-EOT	0.414	0.125	0.269	-0.100	0.900	0.600	0.459	0.115	0.574
Sulfurospirillum	9(S)-HpOTrE	0.571	0.026	0.153	0.400	0.700	0.900	0.519	0.069	0.474
Sulfurospirillum	Tetradecanoic acid	**0.718**	0.003	**0.034**	0.700	-0.500	-0.200	0.307	0.308	0.805
Sulfurospirillum	γ-Linolenic acid	0.550	0.034	0.157	0.700	0.100	-0.100	0.314	0.297	0.805
Sulfurospirillum	13-OxoODE	0.557	0.031	0.155	0.500	0.700	0.600	0.585	0.036	0.459
Sulfurospirillum	cis-11,14-Eicosadienoic acid	0.636	0.011	0.081	0.300	-0.800	0.800	0.239	0.432	0.813

Notes: Values are Spearman's ρ. Pooled correlations were calculated across 15 paired samples (n = 5 per treatment). P values for all 90 pooled microbe–metabolite comparisons were adjusted simultaneously using the Benjamini–Hochberg false discovery rate (FDR) procedure. Treatment-specific coefficients (A ρ, B ρ, and C ρ) are descriptive because each treatment contained only five biological replicates. Treatment-adjusted ρ values were obtained after rank transformation and residualization against treatment indicators; the corresponding P values were also FDR-adjusted across 90 comparisons. NA indicates that a within-treatment coefficient could not be estimated because the microbial taxon showed no variation within that treatment.
